# Cognitive appraisal of environmental stimuli induces emotion-like states in fish

**DOI:** 10.1038/s41598-017-13173-x

**Published:** 2017-10-13

**Authors:** M. Cerqueira, S. Millot, M. F. Castanheira, A. S. Félix, T. Silva, G. A. Oliveira, C. C. Oliveira, C. I. M. Martins, R. F. Oliveira

**Affiliations:** 1 Centro de Ciências do Mar (CCMAR), Universidade do Algarve, Faro, Portugal; 2 Laboratoire Ressources Halieutiques, IFREMER, L’Houmeau, France; 3 ISPA – Instituto Universitário, Lisbon, Portugal; 4 Instituto Gulbenkian de Ciência, Oeiras, Portugal; 5 SPAROS Lda., Área Empresarial de Marim, Lote C, 8700-221 Olhão, Portugal; 6 Champalimaud Neuroscience Programme, Champalimaud Center for the Unknown, Lisbon, Portugal

## Abstract

The occurrence of emotions in non-human animals has been the focus of debate over the years. Recently, an interest in expanding this debate to non-tetrapod vertebrates and to invertebrates has emerged. Within vertebrates, the study of emotion in teleosts is particularly interesting since they represent a divergent evolutionary radiation from that of tetrapods, and thus they provide an insight into the evolution of the biological mechanisms of emotion. We report that Sea Bream exposed to stimuli that vary according to valence (positive, negative) and salience (predictable, unpredictable) exhibit different behavioural, physiological and neuromolecular states. Since according to the dimensional theory of emotion valence and salience define a two-dimensional affective space, our data can be interpreted as evidence for the occurrence of distinctive affective states in fish corresponding to each the four quadrants of the core affective space. Moreover, the fact that the same stimuli presented in a predictable vs. unpredictable way elicited different behavioural, physiological and neuromolecular states, suggests that stimulus appraisal by the individual, rather than an intrinsic characteristic of the stimulus, has triggered the observed responses. Therefore, our data supports the occurrence of emotion-like states in fish that are regulated by the individual’s perception of environmental stimuli.

## Introduction

The interest in the study of emotion in non-human animals dates back to the publication of Darwin’s monograph “The Expression of Emotions in Man and Animals”^1^. However, the fact that human emotions are subjectively experienced as feelings has raised difficulties in defining emotion in animals in objective scientific terms^2^. Nevertheless, other dimensions of emotion, namely the expression of emotion-specific behaviour and accompanying physiological responses, have been documented in many species and a consensus has emerged that animals should express organismic states that index occurrences of value in the environment, regardless of whether these states are consciously experienced^2–5^. These organismic states would be triggered by the value, in terms of potential impact in Darwinian fitness, that an animal ascribes to a stimulus and they would instantiate the ability to respond adaptively to environmental threats (e.g. presence of predators; presence of competitors for resources, such as shelters or territories) and opportunities (e.g. mating partners; food availability; possibility of ascending in social hierarchy). Within this framework, these global organismic states, and their behavioural expression, represent the organism’s experience of reward and threat, and as such they can be seen as similar to human core affect states^4^. The evolution of core affect states (or central emotion states *sensu* ^5^) in animals is plausible as it would provide a way for animals to maximize the acquisition of fitness-enhancing rewards while, simultaneously, minimizing exposure to fitness-threatening punishers. Moreover, these emotion-like states are characterized by general functional properties (i.e. scalability, valence, persistence and generalization) that apply across species and thus make them recognizable and suitable for phylogenetic studies of emotion^5^. Recently, this approach has been used to describe, at the behavioural level, the occurrence of a core affect state of defensive arousal in fruit flies repeatedly exposed to a threat stimulus^6^. Thus, the stage has been set for documenting the occurrence of core affect states across phylogeny and to study how evolutionary conserved are the molecular mechanisms and neural circuits underlying them.

In human research core affect has been conceptualized as a dimensional characterization of the emotion experience along two fundamental underlying dimensions: valence (positive/negative) and intensity (or arousal)^7,8^. Hence, core affect can be represented in a two-dimensional space, which became known as the circumplex model of affect^9^, where these two variable define 4 quadrants (Q): Q1 = Positive affect, high arousal (e.g. happiness); Q2 = positive affect, low arousal (e.g. (relaxed mood); Q3 = negative affect, low arousal (e.g. sadness); Q4 = negative affect, high arousal (e.g. fear). The extension of this model to emotion-like states in animals has been proposed by^4^, who suggested that the axis Q3-Q1 defines a reward acquisition system, with Q1 representing appetitive motivational states that facilitate seeking and obtaining rewards and Q3 representing loss or lack of reward and associated low activity states, whereas the axis Q2-Q4 defines a punishment avoidance system, with Q4 associated to active responses to the presence of threat and Q2 to passive responses to low levels of threat. In humans, non-human primates and rodents, where the neural substrates of emotion have been more extensively studied, these two core affect axes have been associated with different neural mechanisms. Reward acquisition relies on the mesolimbic dopaminergic system, in particular the prefrontal cortex and specific hedonic hotspots located in the ventral striatum (e.g. nucleus accumbens)^10,11^, whereas punishment avoidance has been associated either with the fight-or-flight system (in Q4), or with the behavioural inhibition system (in Q2), with the amygdala playing a central role in either case^2,4^.

In order to create internal emotion-like states that support adaptive physiological and behavioural responses towards ecological threats or opportunities, animals must have evolved perceptual and cognitive mechanisms that identify reliable cues in the environment (i.e. aversive vs. appetitive stimuli, respectively)^12^. When specific environmental cues deterministically predict an appropriate response, these responses can be simple reflexes and fixed action patterns elicited by these cues. However, when environmental complexity and variability increase, single environmental cues may no longer be informative and the evolution of appraisal mechanisms that cognitively assess the presence of threats and opportunities in the environment is predicted^13,14^. According to cognitive theories of emotion, individuals continuously monitor the environment using a set of stimulus evaluation checks (e.g. intrinsic valence, novelty, prediction error, capacity for control) in order to evaluate the valence (positive/negative) and salience (high/low) of detected stimuli, and also assess the available organismal resources to deal with them (i.e. coping mechanisms)^15,16^. The outcome of appraisal translates into an adjustment of the core affective state of the animal to the perceived state of the external environment. Although an integrated study of the different stimulus evaluation checks used by animals is still lacking, empirical evidence for the occurrence of each of these checks has been described in a wide range of animals, from fish to mammals (see^16^ for a recent review).

In this study we have used the Gilthead Sea Bream (*Sparus aurata*) to study if perceived stimulus valence (i.e. appetitive vs. aversive) and salience (i.e. high vs. low) trigger specific behavioural, physiological and brain states, indicative of stimulus-appraisal driven emotion-like states in fish. We have selected this species given its economic importance in European aquaculture, which gives an added value to our results in terms of implications for the assessment of welfare of farmed fish^17^. We have used two stimuli with different intrinsic valences (appetitive: food; aversive: physical constraint) that were presented to the focal individuals in a predictable or unpredictable manner. Predictability was used as a proxy of stimulus salience. The effect of predictability as an appraisal modulator has already been documented in other fish species, both towards aversive and appetitive stimuli^18^. Thus, if emotion-like core affect states are also present in fish we predict that each of the four valence x predictability (salience) treatments will elicit specific brain and physiological states and behavioural profiles, which correspond to each of the four quadrants of the circumplex model of affect described above, namely: Q1 = unpredictable appetitive (UnPRDapp); Q2 = predictable appetitive (PRDapp); Q3 = predictable aversive (PRDavr); Q4 = unpredictable aversive (UnPRDavr).

Brain states for each treatment were characterized using the expression of a set of immediate early genes (see below), as markers of neural activity, in a set of brain regions homologous to those known to be involved in reward and aversion processing in mammals^19^, namely the medial zone of the dorsal telencephalic area (Dm, putative homologue of the mammalian basolateral amygdala); lateral zone of the dorsal telencephalic area (Dl, hippocampus homologue); ventral nucleus of the ventral telencephalic area (Vv, septum homologue)^20,21^. Immediate early genes are expressed in response to external stimuli without requiring previous protein synthesis and act as effector genes, changing the metabolism of the cell, or as transcription factors, orchestrating the cellular profile of gene expression. Consequently, in the field of neuroscience they have been widely used as markers of neuronal activity to map patterns of brain activation in response to specific stimuli or to behavioural tasks^22^. However, it is often the case that different immediate early genes provide different pictures of brain activation^22^, which is most probably due to fact that they are involved in multiple parallel signalling pathways. Thus, we have used the expression of four different immediate early genes [early growth response 1 (*egr-1*), FBJ osteosarcoma oncogene (*c-fos*), brain-derived neurotrophic factor (*bdnf*) and neuronal PAS domain protein 4a *(npas4*)] to characterize central brain states in fish exposed to the different treatments. We have studied the response of each of these genes independently of the others because it is possible that a specific signalling pathway is more related to emotional responses than others, but we have also studied the integrated response of the four genes as an overall neurogenomic state in response to emotional stimuli, as indicated by the patterns of gene co-expression in each brain region. Circulating cortisol levels were used as a marker of the activity of the hypothalamic-pituitary-interrenal axis, which is a major player in the integrated organismal response to environmental stimuli^23^. Finally, behavioural states were characterized by the expression of observed behavioural patterns, namely, social interactions and escape attempts.

## Results

### Appraisal-driven behavioural states

The effectiveness of the predictability treatments (i.e. learning a cue as a predictor of the appetitive or aversive stimuli) on behavioural variables (i.e. escape attempts and social interactions) was validated by showing that relevant behaviours were significantly different between the predictable and unpredictable treatments at the end of the training sessions (see the electronic supplementary material, including Fig. S3, for details). Thus, at the end of the training period the appetitive or aversive stimuli (depending on experimental treatment) became predictable for fish in the predictable treatments, and in the test session individuals were exposed to a predictable/unpredictable aversive or appetitive stimulus, depending on their previous training treatment.

**Figure 1:**
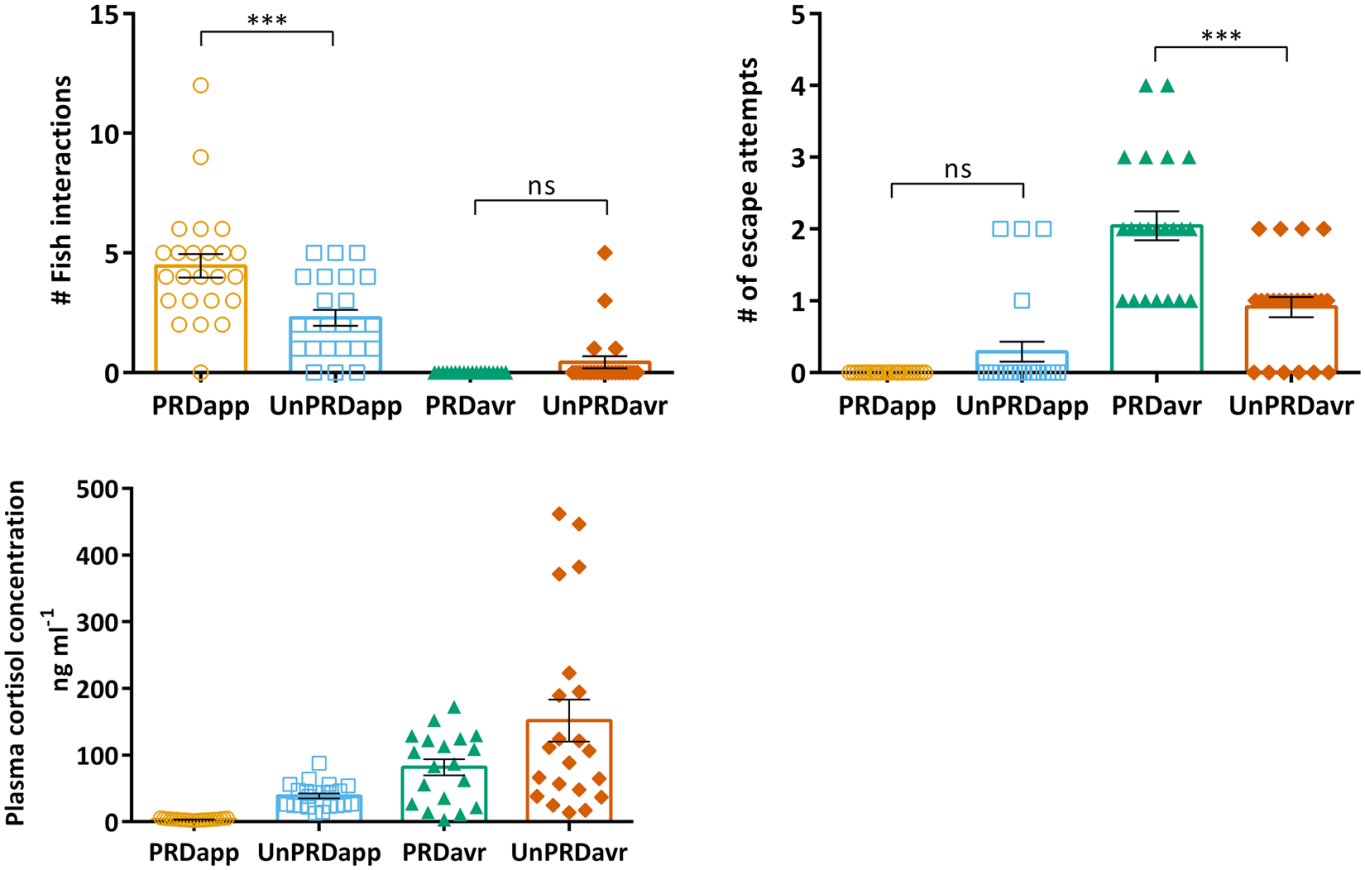
(**a**,**b**) Behaviour expressed by fish during the test session (PRDapp = predictable appetitive treatment; UnPRDapp = unpredictable appetitive treatment; PRDavr = predictable aversive treatment; UnPRDavr = unpredictable aversive treatment): (**a**) frequency of social interactions in the appetitive treatments; (**b**) frequency of escape attempts in the aversive treatments; (**c**) plasma cortisol concentrations measured 30 min after the test session (mean ± SEM). Significant differences between treatments (planned comparisons: PRDapp vs. UnPRDapp; PRDavr vs. UnPRDavr; PRDapp vs. PRDavr and UnPRDapp vs. UnPRDavr) are indicated by asterisks (*p < 0.05; **p < 0.01; ***p < 0.001). All descriptive statistics are mean ± SEM.

The behaviour displayed by fish during the test trial (i.e. exposure to the cue that only predicts experimental stimuli in predictable treatments) was specific of each treatment: expression of social interactions occurred mostly among the individuals of the appetitive treatments (i.e. total of 10 events that have occurred within the same tank in the aversive treatments), and these were more frequent in PRDapp than UnPRDapp (F_(1, 44)_ = 12.21; p = 0.001; see Fig. 1a for planned comparisons); on the other hand, escape attempts were expressed mainly among individuals of the aversive treatments(i.e. total of 7 events in 48 fish), and these were more frequent in PRDavr than in UnPRDavr (valence x predictability: F_(1, 85)_ = 25.61; p < 0.001; see Fig. 1b for planned comparisons). In agreement with these results a significant negative correlation was found between escape events and social interactions (Pearson correlation, R_p_ = −0.393, n = 86, p < 0.001). No effect of the experimental tank of origin was detected for interactions (F_(1, 44)_ = 0.02; p = 0.88) or escape attempts (F_(1, 85)=_1.36; p = 0.25). Thus, appraisal of stimulus valence and predictability elicits specific behavioural profiles (Table 1).

**Table 1:** Linear Mixed Model main effects of stimuli valence and predictability, their interaction and the effect of tank of origin on the behavioral variables (escape attempts and social interactions), cortisol levels and IEG’s mRNA expression in each brain region. Significant values are highlighted in bold.

Variables		**df**		Valence		Predictability		Valence x Predictability		Tank of origin	
Behavior				***F***	***p***	***F***	***p***	***F***	***p***	***F***	***p***
Escape Attempts		1,85		131.77	**<0.001**	5.39	**0.02**	25.61	**<0.001**	1.36	0.25
Fish Interactions		1,85		171.74	**<0.001**	3.87	**0.05**	15.57	**<0.001**	0.99	0.32
Cortisol (ng ml^−1^)		1,50		122.82	**<0.001**	74.76	**<0.001**	28.09	**<0.001**	0.009	0.99
IEGs	**Regions**										
*egr-1*	**Dm**	1,24		0.32	0.57	5.50	**0.03**	0.28	0.60	0.02	0.88
	**Dl**	1,24		0.06	0.81	0.01	0.91	1.93	0.18	1.70	0.20
	**Vv**	1,24		0.15	0.70	23.74	**<0.001**	3.64	0.07	0.02	0.87
*c-fos*	**Dm**	1,24		0.18	0.67	4.48	**0.04**	2.14	0.16	1.11	0.30
	**Dl**	1,24		0.37	0.55	1.18	0.29	0.41	0.53	0.26	0.62
	**Vv**	1,24		0.28	0.60	46.11	**<0.001**	0.49	0.49	0.19	0.67
*bdnf*	**Dm**	1,24		0.24	0.63	2.48	0.13	0.31	0.58	0.49	0.49
	**Dl**	1,24		0.89	0.35	1.61	0.21	0.01	0.91	2.07	0.16
	**Vv**	1,24		25.86	**<0.001**	55.83	**<0.001**	8.89	**0.007**	0.05	0.82
*npas4*	**Dm**	1,24		10.94	**0.003**	1.59	0.22	1.36	0.26	1.58	0.22
	**Dl**	1,24		1.54	0.23	0.19	0.67	0.15	0.70	0.37	0.55
	**Vv**	1,24		1.25	0.27	26.45	**<0.001**	0.42	0.52	0.22	0.64

### Appraisal-driven physiological states

Plasma cortisol levels were affected by both valence (F_(1,50)_ = 122.82; p < 0.001) and predictability of the stimulus (F_(1,50)_ = 74.75; p < 0.001), and an interaction between both experimental factors was also found (valence x predictability: F_(1,50)_ = 28.10; p < 0.001). Under both valences (i.e. appetitive or aversive) fish in unpredictable treatments had higher cortisol levels than in predictable treatments (see Fig. 1c for planned comparisons results). Similarly, under both predictability regimens fish exposed to aversive stimulus had higher cortisol levels than fish exposed to appetitive ones (see Fig. 1c for planned comparisons results). No effect of the experimental tank of origin was detected (F_(1, 50)_ = 0.01; p = 0.92). Finally, throughout the training sessions plasma cortisol levels were positively correlated with escape attempts (R_p_ = 0.322, n = 86, p = 0.003) and negatively correlated with social interactions (R_p_ = −0.654, n = 86, p < 0.001).

### Appraisal-driven brain states

**Figure 2:**
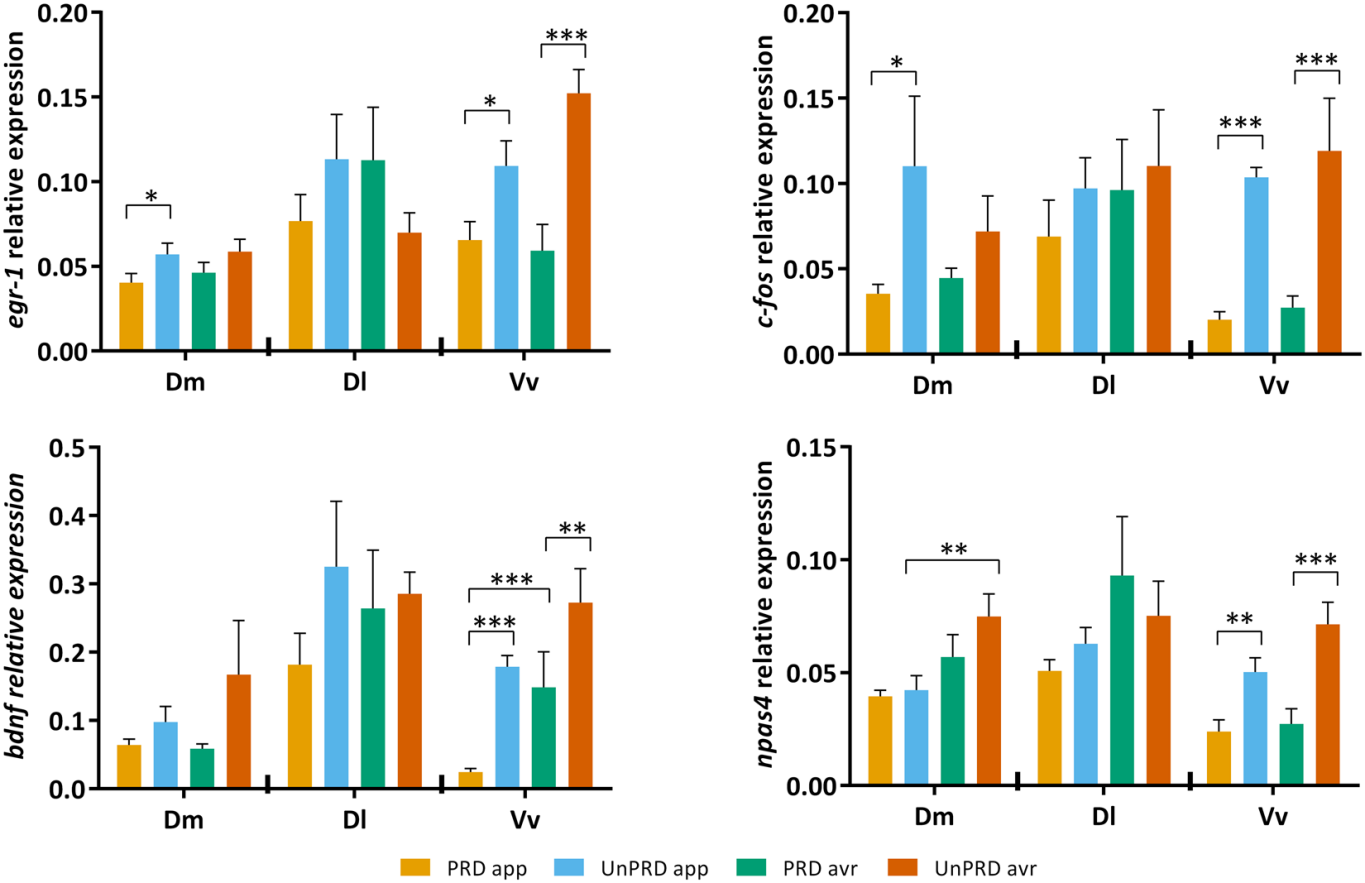
Expression (mean ± SEM) of the immediate early genes *egr-1*, *c-fos*, *bdnf* and *npas4* in the Dm, Dl and Vv brain regions of Sea Bream in the different experimental conditions. Significant differences (planned comparisons) in expression levels between experimental conditions (i.e. PRDapp vs. UnPRDapp; PRDavr vs. UnPRDavr; PRDapp vs. PRDavr and UnPRDapp vs. UnPRDavr) are indicated by asterisks: *p < 0.05; **p < 0.01; ***p < 0.001. All descriptive statistics are mean ± SEM.

A univariate analysis of immediate early gene expression at each of the three candidate brain regions sampled shows that Dl did not respond to either stimulus valence or predictability, whereas both Dm and Vv exhibited changes driven by either valence or predictability. The main effects of stimulus valence were restricted to the expression of *bdnf* in Vv and *npas4* in Dm. On the other hand, predictability had a main effect in the expression of all studied genes (*egr-1*, *c-fos, bdnf, npas4*) in Vv, and also of *egr-1* and *c-fos* expression in Dm (Table 1; Fig. 2). Finally, there was a significant interaction between stimulus valence and predictability in the expression of *bdnf* in Vv (Table 1).

**Figure 3:**
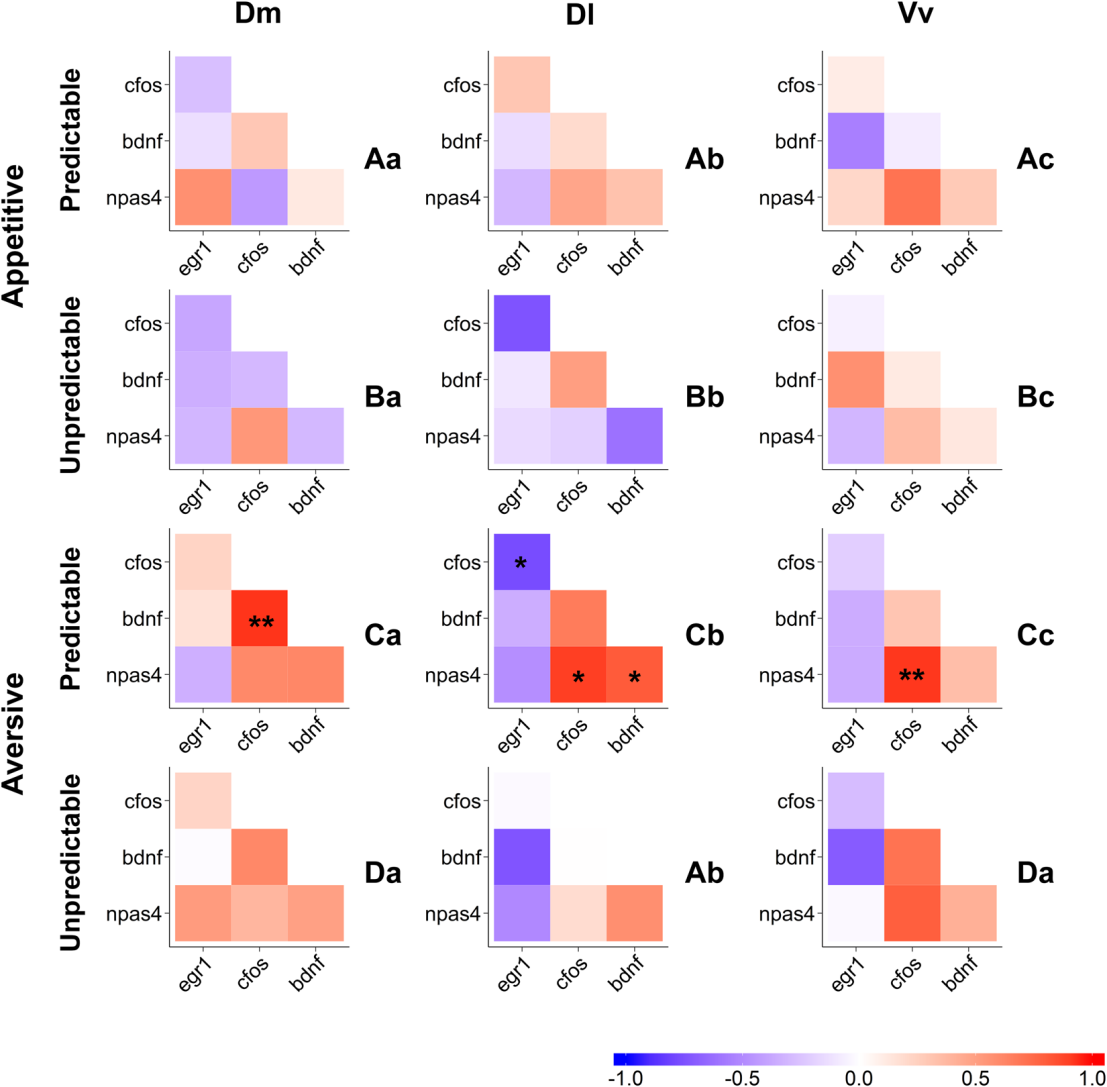
Neurogenomic states, as described by correlation (r) matrices of immediate early genes expression in the different brain nuclei (Dm, medial zone of the dorsal telencephalic area; Dl, lateral zone of the dorsal telencephalic area; Vv, ventral nucleus of the ventral telencephalic area) for each affective state (PRDapp = predictable appetitive treatment; UnPRDapp = unpredictable appetitive treatment; PRDavr = predictable aversive treatment; UnPRDavr = unpredictable aversive treatment); Colour scheme represents r-values from -1 (blue) to 1 (red); Asterisks indicate significant correlations after p-value adjustment: *p < 0.05; **p < 0.01; ***p < 0.001; different capital letters indicate significantly different co-expression patterns among affective states, and different small letters indicate significantly different co-expression patterns among brain nuclei, using the QAP correlation test.

Neurogenomic states, as represented by co-expression matrices of the target genes, across the studied brain regions and across experimental treatments are presented in Fig. 3 (see the electronic Supplementary Material Tables S2 and S3 for detailed information on QAP correlations, used to infer significance differences between pairs of co-expression matrices). The neurogenomic states of Dm and Vv were unique for each of the four experimental treatments (PRDapp, UnPDRapp, PDRavr, UnPRDavr), whereas the neurogenomic state of Dl was similar between PRDapp and UnPRDavr, which were significantly different from either UnPRDapp or PRDavr (Fig. 3). Regarding the comparison of neurogenomic states across brain regions within each treatment, all treatments presented different gene co-expression patterns across all brain regions, except for UnPRDavr in which case Dm and Vv presented similar neurogenomic states (Fig. 3).

### Correlations between appraisal-driven behavioural, physiological and neuromolecular responses

A positive correlation was found between escape attempts and the expression of *bdnf* in the Vv, whereas social interactions were negatively correlated (R_p_ = 0.521, n = 32, p = 0.002; R_p_ = −0.597, n = 32, p < 0.001, respectively). Positive correlations were also found between cortisol levels and mRNA expression in the Dm of *egr-1*and Vv of *c-fos* (R_p_ = 0.434, n = 32, p = 0.013; R_p_ = 0.410, n = 32, p = 0.020, respectively), in the Dm and Vv of *npas4* (R_p_ = 0.356, n = 32, p = 0.046; R_p_ = 0.436, n = 32, p = 0.013, respectively) and in Vv of *bdnf* (R_p_ = 0.747, n = 32, p < 0.001).

### Discrimination of appraisal-driven core affective states based on neuromolecular and physiological data

Stepwise linear discriminant function analysis (LDA) was used to investigate if cortisol levels and immediate early gene expression across candidate brain regions can predict to which of the four combinations of stimulus valence and salience (i.e. predictability), which correspond to the four quadrants of the core affective space, the individuals had been exposed to. The LDA for valence (i.e. PRDapp and UnPRDapp vs. PRDavr and UnPRDavr) revealed a single discriminant function (Wilk’s lambda = 0.477, chi-square = 21.49, p < 0.001), which was significantly loaded with the expression of *npas4* in Dm (0.530) and with cortisol level (0.831), that explained 100% of the variance, hence classifying correctly 100% of the individuals that belong to each valence treatment. Similarly, the LDA for salience (i.e. predictability) (i.e. PRDapp and PRDavr vs. UnPRDapp and UnPRDavr) also revealed a single discriminant function (Wilk’s lambda = 0.170, chi-square = 49.58, p < 0.001), that explained 100% of the variance, hence correctly classifying all the individuals that belong to each salience treatment. This function was significantly loaded with the expression of *egr1* in Dm (0.580) and of *egr1* (0.635), *c-fos* (0.705), and *bdnf* (0.544) in Vv.

**Figure 4:**
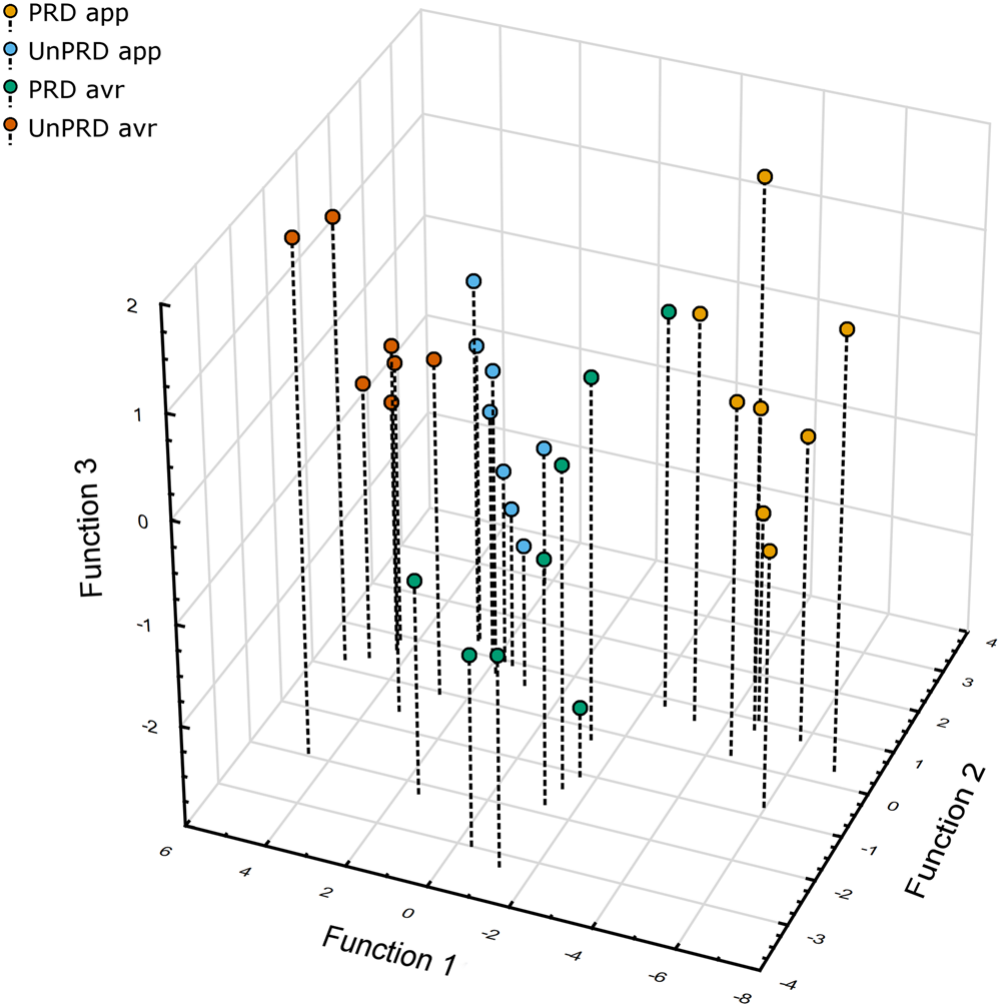
Linear discriminant analysis of the four affective states induced by the four experimental treatments (PRDapp = predictable appetitive treatment; UnPRDapp = unpredictable appetitive treatment; PRDavr = predictable aversive treatment; UnPRDavr = unpredictable aversive treatment) as a function of cortisol and immediate early genes levels in all brain regions. Discriminant scores for each individual are plotted.

When the six variables that were significantly loaded in the valence and salience discriminant functions were used to feed a LDA to predict the valence x salience treatment (as a proxy for affective state), this LDA revealed three significant functions (function 1: Wilk’s lambda = 0.022, chi-square = 98.98, p < 0.0001; function 2: Wilk’s lambda = 0.316, chi-square = 29.94, p < 0.001; function 3: Wilk’s lambda = 0.696, chi-square = 9.43, p = 0.05), with functions 1–3 explaining 89%, 8.1% and 2.9% of the variance respectively (Fig. 4). Analysis of canonical discriminant function coefficients showed that: function 1 was most heavily loaded by *bdnf* and *egr1* expression in Vv (0.856 and 0.646, respectively) and by *egr1* expression in Dm (0.674); function 2 was most heavily loaded by cortisol levels (−0.800) and by *egr1* levels in Dm (0.595); and function 3 was most heavily loaded by *npas4* levels in Dm (0.713). Function 1discriminated between treatments with different salience (i.e. PRDapp and PRDavr from UnPRDapp and UnPRDavr), whereas functions 2 and 3 jointly discriminate between treatments with different valences (i.e. PRDapp and UnPRDapp vs. PRDavr and UnPRDavr) (Fig. 4). Together these three functions allowed the correct classification of all individuals according to their treatment (i.e. affective state).

## Discussion

In this study we have shown that Sea Bream exposed to stimuli that vary according to valence (appetitive, aversive) and salience (predictable, unpredictable) exhibit different behavioural, physiological and neuromolecular states that are specific to each combination of valence and salience (i.e. appetitive predictable, appetitive unpredictable, aversive predictable, aversive unpredictable). At the behavioural level fish exposed to each valence by salience combination exhibited specific behavioural profiles, with appetitive stimuli promoting the occurrence of social interactions and aversive stimuli triggering escape attempts. These behaviours were both more frequent in predictable than in unpredictable treatments. Thus, stimulus valence elicited the expression of qualitatively different behaviours, whereas stimulus salience affected quantitatively behavioural expression. At the physiological level circulating cortisol levels were higher in fish exposed to aversive than to appetitive stimuli, and within each valence unpredictable stimuli elicited higher cortisol levels. Again, stimulus of each valence by salience combination elicited distinct cortisol levels. Finally, in order to characterize central states at the level of the central nervous system we have sampled the expression of a set of immediate early genes involved in experience-driven neuroplasticity, namely *egr1*, *c-fos*, *bdnf* and *npas4*, in three brain regions that are homologues in teleost fish to areas known to be involved in reward and aversion processing in mammals (Dm = amygdala, Dl = hippocampus; and Vv = septum). Two (Dm and Vv) out of these three brain regions showed specific responses to emotional stimuli. Stimulus valence triggered different expression profiles of *bdnf* in Vv and of *npas4* in Dm, with aversive stimuli eliciting higher expression levels of both genes than appetitive stimuli. Stimulus salience (predictability) elicited different expression profiles of all four studied genes in Vv (unpredictable > predictable) and of *c-fos* and *egr1* in Dm (unpredictable > predictable). Thus, overall activity of Vv seems to be associated with stimulus valence. Moreover, neurogenomic states, as capture by the co-expression profile (i.e. correlation matrices) of the four studied genes across the three studied brain regions, were unique for each of the four experimental treatments, suggesting that stimulus of each valence by salience combination elicited distinct central states. Finally, we have used linear discriminant function analyses to check if the measured behavioural, physiological and neuromolecular variables could efficiently discriminate the four experimental treatments. A discriminant function that correctly classified 100% of individuals according to stimulus valence that they have been exposed to was significantly loaded with the expression of *npas4* in Dm and with cortisol levels, whereas another discriminant function that classified correctly 100% of the individuals belonging to each salience treatment was loaded with the expression of *egr1* in Dm and of *egr1*, *c-fos*, and *bdnf* in Vv. The variables that were significantly loaded in these two discriminant functions were then used to develop a third discriminant analysis aiming at discriminating the four experimental treatments, which correctly classified 100% of individuals according to treatment. Thus, physiological and neurogenomic state can successfully discriminate the four combinations of valence by salience stimuli. Since according to the dimensional theories of emotion^4,7^ valence and salience define a two-dimensional affective space, our data can be interpreted as evidence for the occurrence of distinctive affective states in fish corresponding to each the four quadrants of the core affective space.

Emotions have been described as internal brain states associated with expressive behaviours, which humans experience as feelings^3^. Since animals cannot report the subjective experience of feelings the assessment of emotional states in animals has to rely on the occurrence of specific behaviours associated with internal central states. Thus, from a comparative perspective an emotion can be defined as a brain state, encoded by the activity of specific neural circuits, that is triggered by specific stimuli and that elicits the expression of specific behaviours and other external cues^5^. From this perspective, the results reported here showing that external stimuli of different valence and salience triggers the expression of specific behavioural profiles associated with specific physiological and neuromolecular states supports the occurrence of emotion-like states in fish. Given that emotional states are often associated with human behaviour this result may sound surprising at first. However, the evolution of affective states (i.e. emotions/mood) in animals has been predicted by theoretical models of adaptive decision-making, since it allows an adjustment of the response to cues of reward and punishment according to the autocorrelation of aversive and appetitive events in the environment and internal condition, rather than using a fixed response threshold. Thus, the modulation of decision-making by core affective states would allow animals to give more efficient responses to a wide range of fitness threatening and fitness enhancing events^24–27^. More recently, it has been proposed that these affective states share a number of properties, namely scalability, valence, persistence and generalization, which have been named emotion primitives, that allow their recognition in phylogenetically distant organism, hence opening the study of the biological mechanisms of emotion across different taxa^5,6^. In a previous study using a conditioned place preference/avoidance paradigm we have shown that Sea Bream exposed to appetitive or aversive stimuli have valence-specific responses (preference vs. avoidance, respectively) that are persistent in time, even when only the CS (i.e. conditioned place) is present^28^. Thus, in Sea Bream, at least two of these emotion primitives are present.

The fact that in this study the same stimulus presented in a predictable vs. unpredictable way elicited different behavioural, physiological and neuromolecular states suggests that stimulus appraisal by the individual, rather than an intrinsic characteristic of the stimulus, such as its valence, is triggering the observed responses. Therefore, the occurrence of emotion-like states in fish seems to be regulated by the individual’s perception of environmental stimuli. The role of cognitive appraisal in the regulation of stress and emotional states was first proposed in humans and has subsequently been expanded to other animals^4,15,16^. In fish the occurrence of cognitive appraisal has been documented in different species(e.g.^18,28–31^; however, its neural bases have not been investigated yet in fish and the present study provided a new insight into these mechanisms.

Molecular markers of neuronal activity, such as the expression of immediate early genes, have been used to characterize behaviourally relevant global brain states, with high spatial resolution^32^. An assumption used in this approach is that behavioural states can be mapped into neuromolecular states of relevant brain regions (aka neurogenomic states,^32,33^). In this study, a similar approach has been used and the occurrence of specific neurogenomic states induced by emotional stimuli has been investigated in a set of brain regions in Sea bream that are putative homologues of regions involved in emotional stimuli processing in mammals, namely the amygdala (Dm), the lateral septum (Vv) and the hippocampus(Dl)^25,34^. In mammals the prefrontal cortex is also known to play an important role in emotional regulation^35^, but a homologue area in teleost fish is not known. The four immediate early genes used (i.e. *egr-1*, *c-fos*, *npas4* and *bdnf*) are involved in different signalling pathways and thus were expected to capture complementary information on neural activation^36–38^. Indeed, these genes exhibited different expression profiles. However, all four genes exhibited a similar pattern of expression in the Vv, with predictability of either appetitive or aversive stimuli heightening their mRNA levels. Moreover, the linear discriminant analysis for predictability identified a significant function loaded with the expression of *egr-1*, *c-fos* and *bdnf* in Vv and *egr-1* in Dm. Thus, Vv seems to play a key role in the appraisal of stimulus predictability in fish. This result is in line with the role of the lateral septum in the perception of stimulus novelty and emotional regulation in other species^34,39,40^. In mammals the septum also establishes a circuit with hippocampus (i.e. septo-hippocampal pathway) that modulates memory formation and recall in the hippocampus^41^. Thus, the increased activation of Vv in fish of predictable treatments may also reflect associative learning of the CS that signalled the aversive/appetitive stimulus in our experiment. On the other hand, the expression of *bdnf* in Vv and of *npas4* in Dm was higher in the aversive treatments and the discriminant function for stimulus valence was significantly loaded with the expression of npas4 in Dm and with cortisol levels, hence suggesting an involvement of Dm, eventually modulated by cortisol levels, on the appraisal of stimulus valence. Again this result is in line with results from other studies in mammals that have shown an involvement of the basolateral amygdala in responses to emotional stimuli^42^ and of *npas4* in fear memory^38,43^. Thus, the neuromolecular data presented here suggest an involvement of both Vv and Dm in the appraisal of emotional stimuli, which supports the occurrence of an evolutionary conserved neural substrate for the processing of emotional stimuli, given the similar role played by the mammalian homologues of these areas.

## Methods

All experimental procedures were conducted in accordance with the Guidelines of the European Union Council (86/609/EU) and the Portuguese legislation for the use of laboratory animals and were approved by Portuguese licensing authority for animal experimentation (Direção Geral da Alimentação e Veterinária, Portugal; permit number 0420/000/000-n.99-09/11/2009).

### Subjects and maintenance

Fish [initial body weight = 39.45 g ± 10.39 g (mean ± SD)] were housed in fibre glass tanks (500 L) at Ramalhete Research Station (University of Algarve, Faro, Portugal), provided with constant aeration and set in an open water circuit, under the following rearing conditions during 3 months before the start of the experiment: temperature = 21 ± 3 °C; salinity = 35 ± 2%; dissolved oxygen >75%; 12 L: 12D photoperiod, with lights on at 08:00 h; density of 2 kg m^−^³; daily feeding of 3% of body weight of a commercial diet (Aquagold 3 mm, Aquasoja)].

### Experimental procedures

Twelve experimental aquaria (70 × 40 × 30 cm) in an open water circuit were used, under the same housing conditions as described above for the stock tanks Fish were fed twice a day at 1.5% BW. Inside each aquaria, covering all the bottom area, there was a blue plastic basket of approximately 60 l (64 cm length × 38 cm width and 25 cm depth), attached to a lifting mechanism (see Fig. S1). The sides of the experimental aquaria were covered with opaque plastic in order to prevent visual contact between the focal animals and the experimenters.

Individuals (N = 96) were measured, weighted, tagged (Floy Tag Manufacturing Inc, Seattle, USA) and then divided into groups of 4 individuals each. Six groups were tested for each experimental treatment (see below; n = 24 fish/treatment). The experiment lasted 15 days, the first 12 corresponding to the acclimation period and the last 3 to the experimental period. Fish were exposed to 8 training sessions (4 training sessions/day on the first 2 days of the experimental period) followed by 1 test session (morning of day 3), using a delay-conditioning protocol, with light (12 V, 25 W) as a conditioned stimulus (CS), food as the appetitive unconditioned stimulus, and air exposure as the aversive unconditioned stimulus (USapp and USavr, respectively). Four training treatments were used: (1) Predictable appetitive treatment (PRDapp) - Groups of 4 fish were trained to associate the turn on of the light during 2 min (CS) with a subsequent food reward (USapp: food pellet dropped each 2 sec during the last minute of the light on); (2) Predictable aversive treatment (PRDavr) – Similar to PRDapp but the CS used was air exposure (USavr: lifting the basket from the aquarium in approximately 5 sec during the last minute of the light on period); (3) Unpredictable appetitive treatment (UnPRDapp) - Groups of 4 fish were randomly exposed to a food reward (USapp) not coinciding with the CS (i.e. CS presented either 30 min before or 30 min after US); (3) Unpredictable aversive treatment (UnPRDavr) – Similar to UnPRDapp but the CS used was air exposure (USavr). On the test session all groups were tested in the presence of US + CS.

### Behavioural observations

The behaviour of fish was video recorded (top view) both during the training and in the test sessions. Videos were subsequently analysed using a multi-event recorder software (Observer XT®, Noldus, Netherlands). The response of fish to the CS was assessed by: (1) social interactions - frequency of frontal and lateral displays, chases or flees; and (2) escape behaviour – frequency of escape attempts through the holes on the plastic basket. All videos were coded by a single observer, which was blind to the experimental treatment during video-analysis.

### Blood Sampling and plasma cortisol analysis

Thirty min after the test session, fish were caught and euthanized with an overdose of 2-phenoxyethanol (1%, Sigma-Aldrich). Blood was immediately collected and centrifuged for 15 min at 2000 g and plasma was frozen and stored at −80 °C until further processing. Plasma cortisol levels were measured using a commercial ELISA kit (RE52061, IBL Hamburg, Germany), with a sensitivity of 2.5 ngml^−1^, which has been previously validated for Sea Bream^44^. Intra- and inter-assay coefficients of variation were 2.9% and 3.5%, respectively.

### Brain microdissection and gene expression analysis

Eight individuals from each experimental treatment were randomly selected for the assessment of immediate early gene mRNA expression. After sacrifice (see above) the skull, with the brain inside, was removed from the fish, embedded in Tissue-Tek®, and kept at −80 °C until further processing. Brain telencephalon slices were obtained through 150 µm thick cryostat (Leica, CM 3050 S) coronal sections. The following brain regions, identified according to^45^, were then microdissected (see supplementary material and Fig. S2 for detailed description): medial part of the dorsal telencephalon (Dm), lateral telencephalon (Dl) and ventral nucleus of the ventral telencephalon (Vv). Tissue was collected directly into lysis buffer from Qiagen Lipid Tissue Mini Kit (#74804; Valencia, CA), total RNA extracted from the samples, reverse transcribed to cDNA (BioRad iScript cDNA Synthesis Kit; Valencia, CA), and used as a template for quantitative polymerase chain reactions (qPCR) of *egr-1*, *c-fos*, *bdnf* and *npas4*. The geometric mean of the expression of two previously established housekeeping genes, *eef1a* and *18S*was used as an internal control (see the electronic supplementary material for primer sequences and qPCR conditions).

### Statistical analysis

Descriptive statistics are expressed as mean ± standard error of the mean (SEM). The assumptions of normality and homoscedasticity were confirmed by analysis of the residuals. Homogeneity of variance was checked by Levene’s test and transformation of variables was used [log for cortisol and gene expression variables, and log (x + 1) for behavioural variables] to achieve homogeneity.

Linear mixed model (LMM) analyses were used to assess the effect of each experimental factor (i.e. stimuli valence: app vs. avr; and predictability: PRD vs. UnPRD) on the behavioural variables in the test trial, on cortisol levels and on IEGs mRNA expression (*egr-1*, *c-fos*, *bdnf* and *npas4*) in each brain region (Dm, Dl and Vv). Given that we have used more than one individual from the same experimental tank in each treatment, pseudo replication concerns could be raised. In order to match this design, individuals were nested within the experimental tank of origin and used as a random effect in each LMM. Moreover, including the tank of origin as a fixed factor, we did not find an effect of this variable on the experimental treatments (see results).

All LMM were estimated using the restricted maximum likelihood method. A priori planned comparisons were used to test for specific differences between experimental conditions, namely: PRDapp vs. UnPRDapp; PRDavr vs. UnPRDavr; PRDapp vs. PRDavr; and UnPRDapp vs. UnPRDavr. Pearson test was used to depict correlations among behavioural variables, between behaviour and cortisol, and between those and gene expression.

Stepwise linear discriminant analyses (LDA), were used to determine which measures of physiological (i.e. cortisol) and central state (i.e. immediate early genes expression in different brain nuclei) are the best predictors of affective states. The F statistic was used as a measure of the contribution of each variable (cortisol concentration and IEG expression in each brain region) to the discriminant functions. An F-value above 3.84 was used as the selection criteria for predictors to enter the model and predictors were removed when the F-value dropped below 2.71 (e.g.^46^). First, two LDA were run to discriminate between treatments with different valence (i.e. PRDapp and UnPRDapp vs. PRDavr and UnPRDavr) and salience (i.e. PRDapp and PRDavr vs. UnPRDapp and UnPRDavr), independently. Next, the variables that better discriminated valence and salience, independently of each other, were used to feed a third LDA for the four experimental treatments (PRDapp, UnPRDapp, PRDavr, UnPRDavr). Factor loadings above 0.30 were considered important for interpreting discriminant functions.

The neurogenomic states, as indicated by the patterns of gene co-expression in each brain region, elicited by each experimental treatment were represented using heatmaps of Pearson correlations matrices, with p-values adjusted following the Benjamini and Hochberg’s method^47^. Differences in gene co-expression patterns between brain areas within each experimental condition, and between experimental conditions within each brain area, were assessed using the quadratic assignment procedure (QAP) correlation test with 5000 permutations^48^. The null hypothesis of the QAP test is that when p > 0.05 there is no association between matrices, hence a non-significant p-value indicates that the correlation matrices are different.

The LMM, planned comparisons and QAP correlations performed to evaluate the neurogenomic states were run on R® (R Development Core Team). LDA statistical procedures were run on IBM SPSS® statistics v19.0 and GraphPad Prism® v6.0 for windows was used for chart building and figures layout.

## Electronic supplementary material

Supplementary information
