# Supplementary material for: Cognitive appraisal of environmental stimuli induces emotion-like states in fish
Source: Sci Rep. 2017 Oct 13;7:13181. doi: 10.1038/s41598-017-13173-x (PMC5640617; doi:10.1038/s41598-017-13173-x)
Supplement: Supplementary file 1 — Supplementary information [file 41598_2017_13173_MOESM1_ESM.pdf]

Electronic Supplementary Material

Cognitive appraisal of environmental stimuli induces emotion-like states in fish

M. Cerqueira, S. Millot, M.F. Castanheira, A. S. Félix, T. Silva, G. A. Oliveira, C.C. Oliveira, C.I.M. Martins, R.F. Oliveira

Correspondence to:

Rui F. Oliveira,
Integrative Behavioural Biology
Instituto Gulbenkian de Ciência,
Rua da Quinta Grande 6,
2780-156 Oeiras, Portugal
ruiol@ispa.pt

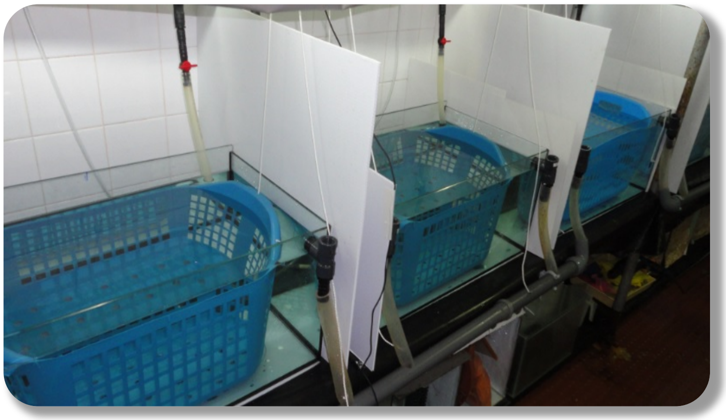

Figure S1. Experimental set-up used in the research driven to assess the effect of predictability and stimulus valence on fish stress response.

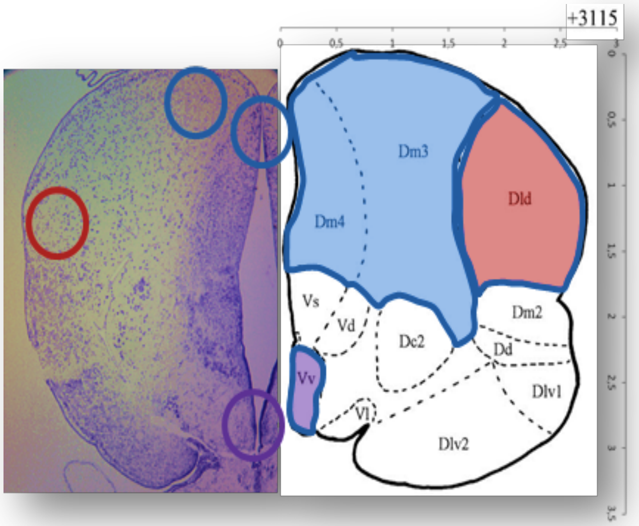

Figure S2. Coronal sections of Gilthead seabream (*Sparus aurata)* telencephalon spaced 400 µm from each other. Nissl staining images (left) and an illustrative section (right) adapted from [1] highlighting the areas of interest from the telencephalon. Areas of interest: medial part of the dorsal telencephalon (Dm, Blue), lateral telencephalon (Dl, red), ventral nucleus of the ventral telencephalon (Vv, purple).

Detailed Methods

Validation of the predictability treatments

Fish in the predictable treatments (PRDapp and PRDavr) were trained (8 training trials) to associate a conditioned stimulus (CS) with an unconditioned stimulus (US) of either positive (US = food reward) or negative (US = physical confinement) valence. The analysis of the behavioural patterns throughout the training sessions indicates that fish in the predictable treatments (PRDapp and PRDavr) established an association between CS and US, as indicated by significant differences in: (1) social behaviour on trial number between UnPRDapp and PRDapp (Fig.S3a; Predictability main effect: $F(1,44)=10.46$, $p=.002$; Predictability x Session: $F(7, 308)=8.76$, $p<.001$; Tank effect: $F(1,44)=2.56$, $p=.12$); and (2) escape attempts on trial number between UnPRDavr and PRDavr (Fig.S3b; Predictability main effect: $F(1,41)=40.94$, $p<.001$; Predictability x Session: $F(7, 287)=5.88$, $p<.001$; Tank effect: $F(1,41)=1.13$, $p=.29$). Thus, social behaviour, in the case of appetitive treatments and escape attempts in the case of aversive treatments, increased significantly more with training in the predictable situations.

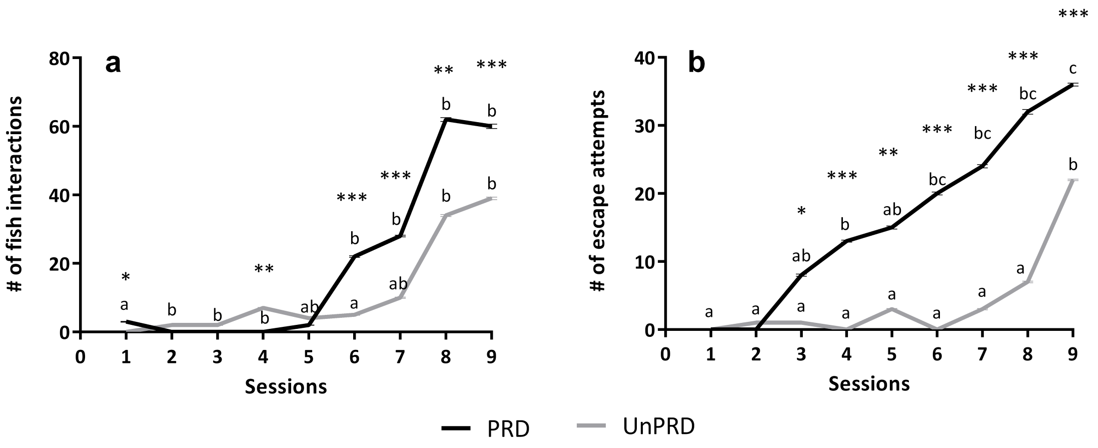

Figure S3. Behavioural and physiological responses to stimuli of different valence and salience (i.e. predictability). (a-b) Behaviour expressed by fish during the training sessions for predictability (PRD = predictability treatment; UnPRD = unpredictability treatment): (a) frequency of social interactions in the appetitive treatments;; (b) frequency of escape attempts in the aversive treatments; significant differences (post-hoc tests) across trials within the same treatment are indicated by different letters; significant differences (planned comparisons) between treatments at each trial are indicated by asterisks (* $p < 0.05$; ** $p < 0.01$; *** $p< 0.001$).

Brain microdissection and gene expression analysis

Eight individuals from each experimental treatment were randomly selected for the assessment of immediate early gene mRNA expression. After sacrifice (see above) the skull, with the brain inside, was removed from the fish, embedded in Tissue-Tek®, and kept at -80 ºC until further processing. Brain telencephalon slices were obtained through 150 µm thick cryostat (Leica, CM 3050S) coronal sections. The microdissections were performed with modified 25G steel needles in different regions of the telencephalon: medial part of the dorsal telencephalon (Dm), lateral telencephalon (Dl) and ventral nucleus of the ventral telencephalon (Vv) (see detailed description in Fig. S2). Brain areas were identified and classified according to [1]. Tissue was collected directly into lysis buffer from Qiagen Lipid Tissue Mini Kit (#74804; Valencia, CA) and total RNA extracted from the samples (see below).

Rna extraction (adapted from previous works developed in our laboratory)

Tissue was homogenized in quiazol lysis reagent by vortex followed by an incubation of 7 min at room temperature (RT). It was added chloroform in a proportion of 1:2 and the sample incubated at RT for 5 min. Samples were then centrifuge at 13000 g for 20 min at 4 ºC, and the upper aqueous phase transferred to new tube where 1 volume of 70 % ethanol was added. This mixture was transferred to an RNEasy column, remained 5 min at RT, being subsequently centrifuged for 1 min at 9000 g. A sequence of buffers was added to the Rneasy column according to the manufacturer's instructions, and RNA eluted with 25 µl of RNAse-free water. Each sample was then taken to Nanodrop to assess the "quality" of the RNA.

Primers design and quantitative real-time PCR (qPCR)

The qRT-PCR protocol was based and adapted on procedures previously performed by [2]. Partial sequences for *18S* (accession # AM490061.1), *eef1a* (accession # AF184170.1), *egr-1* (accession # KC442101.1) and *c-fos* (accession #GU108576.1), were obtained from the National Center for Biotechnology Information (NCBI, http://www.ncbi.nlm.nih.gov/nuccore). For *bdnf* and *npas4*, primers were designed using NCBI sequences from several fish species and then aligned with ClustalW to select the most conserved regions (www.genome.jp/tools/clustalw) [3]. Primers for all target mRNA were designed using Primer3 software [4,5] and synthetized by Sigma-Aldrich (Hamburgo, German). The PCR products were sequenced to confirm the desired primer cDNA amplification. Primer dimers formation was controlled with FastPCR v5.4 software [6] and optimal annealing temperature was assessed for maximal fluorescence (see Table 3.1.2). Fluorescence cycle thresholds (CT) were automatically measured using a Roche Light Cycler 480 II (Roche Diagnostics, Penzberg, Germany), and relative expression of the target genes were calculated using the $2^{-\Delta Ct}$ method (Livak and Schmittgen, 2001). The qRT-PCR was performed using 25 µL reactions including 12.5 µL Light cycler H 480 SYBR Green I Master (Roche diagnostics GmbH, Mannheim, Germany), 0.2 µL of each primer and 1 µL of cDNA template (RNA equivalent). Cycling conditions were as follows: (i) denaturation (5 min at 95 °C); (ii) amplification and quantification (40 cycles; 30 s at 95 °C, 45 s at primer specific annealing temperature (see table S2 for details), 30 s at 72 °C with a single fluorescence measurement); and (iii) melting curve assessment (30 s at 95 °C; 30 s at 55 °C, followed by an 55-95 °C with a heating rate of 0.5 °C/s and a continuous fluorescence measurement; 30 s at 95 °C). All reactions were run in duplicate and controls without DNA templates were run to verify the absence of cDNA

contamination. Primers efficiency was calculated for each qRT-PCR reaction using Light Cycler 480 II inner software.

Table S1 |List of primers and respective temperature of annealing used for quantitative real-time PCR from the different telencephalon regions studied in Gilthead seabream (*Sparus aurata)* brain.

Gene	Forward Primer	Reverse Primer	Ta*
**18S[1]**	AGGGTGTTGGCAGACGTTAC	CTTCTGCCTGTTGAGGAACC	57
**EeF1a[1]**	TGGCTTCAACATCAAGAACG	ATGTGAGCTGTGTGGCAATC	57
**c-Fos[2]**	GGAGAGGAAGCAACAGCAAC	TACAAGTCCGCTCCATAGCC	58
**EGR-1[2]**	ACCTGTGATCGTCGCTTCTC	ATCCTTCTGCCGTAAGTGGA	58
**BDNF[2]**	GCTCAGCGTGTGTGACAGTA	ACAGGGACCTTTTCCATGAC	59
**NPAS4[2]**	CAACCAAAGGAGCATCCAAG	AGCCGTGCTTTATCTGCATC	56

*Ta = Temperature of annealing; [1]Reference genes; [2]Target genes.

Table S2 |Quadratic assignment procedure (QAP) correlation test for the different brain nuclei. Dm, medial zone of the dorsal telencephalic area; Dl, lateral zone of the dorsal telencephalic area and Vv, ventral nucleus of the ventral telencephalic area; between experimental conditions (PRDapp – Predictable appetitive; UnPRDapp – Unpredictable appetitive; PRDavr – Predictable aversive; UnPRDavr – Unpredictable aversive). Significant correlations are indicated in bold for p < 0.05.

	Brain nuclei		
**Experimental conditions**	**Dm**	**Dl**	**Vv**
**PRDapp-UnPRDapp**	r = -0,537<br>p = 0,167	r = -0,266<br>p = 0,302	r = -0,315<br>p = 0,118
**PRDapp-PRDavr**	r = -0,295<br>p = 0,207	r = 0,600<br>p = 0,207	r = 0,707<br>p = 0,164
**PRDapp-UnPRDavr**	r = 0,597<br>p = 0,174	**r = 0,841**<br>**p = 0,044**	r = 0,711<br>p = 0,165
**UnPRDapp-PRDavr**	r = 0,287<br>p = 0,326	r = 0,314<br>p = 0,215	r = 0,305<br>p = 0,161
**UnPRDapp-UnPRDavr**	r = 0,093<br>p = 0,557	r = -0,311<br>p = 0,209	r = -0,097<br>p = 0,333
**PRDavr-UnPRDavr**	r = 0,318<br>p = 0,288	r = 0,692<br>p = 0,200	r = 0,872<br>p = 0,081

Table S3 |Quadratic assignment procedure (QAP) correlation test for the different experimental conditions (PRDapp – Predictable appetitive; UnPRDapp – Unpredictable appetitive; PRDavr – Predictable aversive; UnPRDavr – Unpredictable aversive). Dm, medial zone of the dorsal telencephalic area; Dl, lateral zone of the dorsal telencephalic area and Vv, ventral nucleus of the ventral telencephalic area. Significant correlations are indicated in bold for p < 0.05.

	Experimental conditions			
**Brain nuclei**	**PRDapp**	**UnPRDapp**	**PRDavr**	**UnPRDavr**
**Dm-Dl**	r = -0,649<br>p = 0,08	r = 0,07<br>p = 0,365	r = 0,791<br>p = 0,247	r = 0,523<br>p = 0,167
**Dm-Vv**	r = -0,180<br>p = 0,374	r = 0,335<br>p = 0,248	r = 0,735<br>p = 0,121	**r = 0,796**<br>**p = 0,038**
**Dl-Vv**	r = 0,572<br>p = 0,207	r = 0,170<br>p = 0,504	r = 0,893<br>p = 0,130	r = 0,717<br>p = 0,125

References

1 Munoz-Cueto, J. A., Sarasquete, C., Zohar, A. H. & Kah, O. An atlas of the brain of the gilthead seabream (Sparus aurata). *College Park: Maryland Sea Grant* (2001).

2 Desjardins, J. K. & Fernald, R. D. What do fish make of mirror images? *Biology Letters* **6**, 744-747, doi:10.1098/rsbl.2010.0247 (2010).

3 Thompson, J. D., Higgins, D. G. & Gibson, T. J. CLUSTAL W: improving the sensitivity of progressive multiple sequence alignment through sequence weighting, position-specific gap penalties and weight matrix choice. *Nucleic Acids Res* **22**, 4673-4680 (1994).

4 Koressaar, T. & Remm, M. Enhancements and modifications of primer design program Primer3. *Bioinformatics (Oxford, England)* **23**, 1289-1291, doi:10.1093/bioinformatics/btm091 (2007).

5 Untergasser, A. *et al.* Primer3--new capabilities and interfaces. *Nucleic Acids Res* **40**, e115, doi:10.1093/nar/gks596 (2012).

6 Kalendar, R., Lee, D. & Schulman, A. H. FastPCR software for PCR, in silico PCR, and oligonucleotide assembly and analysis. *Methods in molecular biology (Clifton, N.J.)* **1116**, 271-302, doi:10.1007/978-1-62703-764-8_18 (2014).
